# Supplementary material for: Imaging breast malignancies with the Twente Photoacoustic Mammoscope 2
Source: PLoS One. 2023 Mar 2;18(3):e0281434. doi: 10.1371/journal.pone.0281434 (PMC9980787; doi:10.1371/journal.pone.0281434)
Supplement: S1 Table — ‘NST’ = non-specific type, ‘DD’ = differential diagnosis, ‘oc’ = o’clock. Subjects with IDs 1 through 4 are discussed in detail in this work. aDiagnosis of malignant lesions was based on histopathological examination of biopsy specimens. For cases studied in detail in this work, results of histology of lumpectomy or mastectomy were also obtained, but not included in this table. (DOCX) [file pone.0281434.s001.docx]

**Supporting Information**

**S1 Table.** **Subject characteristics.**

| **ID** | **Age** | **Affected breast(s)** | | **Diagnosis of suspect lesion^a^** | **Location of lesion** | **Estimated size of lesion (mm)** | **MR available? (Y/N)** |
| --- | --- | --- | --- | --- | --- | --- | --- |
|  |  | L | R |  |  |  |  |
| 1 | 58 | L |  | Invasive carcinoma NST grade 1 DD tubular carcinoma, multicentric | 11 – 12 oc | 19 | Y |
| 2 | 79 |  | R | Invasive carcinoma NST grade 3 | 6 oc | 39 | Y |
| 3 | 78 | L |  | Intraductal papillary carcinoma (uncertain) | 9 oc | 33 | N |
| 4 | 56 | L |  | Invasive carcinoma NST grade 3 | 4 oc | 25 | Y |
| 5 | 64 |  | R | Adenosis and papillomatosis | 12 oc | 3 and 6 | Y |
| 6 | 48 |  | R | Cyst | 9 oc | >50 | N |
| 7 | 52 |  | R | Invasive carcinoma NST grade 2 | 12 oc | 8 | N |
| 8 | 42 |  | R | Abscess | 4 oc | 12 | N |
| 9 | 50 | L | R | (L) Invasive carcinoma NST grade 2 with metastasis in axilla;  (R) Fibroadenoma | (L) 2 oc (R) 9 oc | (L) 35 (R) 10 | Y |
| 10 | 59 | L |  | Invasive carcinoma NST grade 2 with metastasis in axilla | Cranial and lateral | >50 | N |
| 11 | 40 | L |  | Invasive carcinoma NST grade 2 | 2 oc | 16 | N |
| 12 | 47 | L |  | Fibroepithelial lesion | 3 oc | 18 | N |
| 13 | 63 | L |  | Invasive carcinoma NST grade 2 | 4 oc | 13 | N |
| 14 | 44 |  | R | Invasive multicentric carcinoma NST grade 3 with metastasis in axilla | Lateral | 60 | Y |
| 15 | 44 | L | R | Multiple cysts in both breasts | - | 50 (palpable lesion) | N |
| 16 | 41 | L | R | Multiple cysts in both breasts | - | 36 (largest lesion) | N |
| 17 | 71 | L |  | Adenosis with metaplasia | 1 oc | 7 | N |
| 18 | 55 |  | R | Invasive carcinoma NST grade 2 | 12 oc | 13 | N |
| 19 | 51 | L |  | Invasive carcinoma NST grade 1 | 4 oc | 28 | Y |
| 20 | 53 |  | R | 3 types of malignant lesions, multicentric | 11 – 3 oc | 10 per lesion | Y |
| 21 | 70 | L |  | Invasive carcinoma NST grade 1 | 8 oc | 7 | N |
| 22 | 48 |  | R | Cyst | 3 oc | 40 | N |
| 23 | 47 | L |  | Cyst | 3 oc | 18 | N |
| 24 | 47 | L |  | Slightly inflamed cyst | 8 oc | 14 | N |
| 25 | 57 |  | R | Invasive carcinoma NST grade 1 | 4 oc | 26 | Y |
| 26 | 46 |  | R | Invasive multicentric carcinoma NST grade 1 | 3 oc | 50 (main lesion) | Y |
| 27 | 56 | L |  | Infiltrating lobular carcinoma grade 1 | 3 oc | 22 | Y |
| 28 | 57 |  | R | Cysts | 8 oc | 12 | N |
| 29 | 30 | L | R | Multiple cysts in both breasts | - | - | N |
| 30 | 48 | L |  | Invasive carcinoma NST grade 3 | 2 oc | 40 | Y |

‘NST’ = non-specific type, ‘DD’ = differential diagnosis, ‘oc’ = o’clock. Subjects with IDs 1 through 4 are discussed in detail in this work.

^a^Diagnosis of malignant lesions was based on histopathological examination of biopsy specimens. For cases studied in detail in this work, results of histology of lumpectomy or mastectomy were also obtained, but not included in this table.

**S1 Fig.** Case 1: Comparison of photoacoustic with conventional MR images. (A) Photoacoustic maximum intensity projections (MIPs) in two planes (coronal (top) and transverse (bottom)), at two illumination wavelengths. (B) Post-contrast dynamic T1 MR MIPs in the same two planes. All scale bars represent 20 mm.

**S2 Fig.** Case 2: Comparison of photoacoustic with conventional MR images. (A) Photoacoustic maximum intensity projections (MIPs) in two planes (coronal (top) and transverse (bottom)), at two illumination wavelengths. (B) Post-contrast dynamic T1 MR MIPs in the same two planes. All scale bars represent 20 mm.

**S3 Fig.** Case 3: Photoacoustic maximum intensity projections (MIPs) in two planes (coronal (top) and transverse (bottom)), at two illumination wavelengths. All scale bars represent 20 mm.

**S4 Fig.** Case 4: Comparison of photoacoustic with conventional MR images. (A) Photoacoustic maximum intensity projections (MIPs) in two planes (coronal (top) and transverse (bottom)), at two illumination wavelengths. (B) Post-contrast dynamic T1 MR MIPs in the same two planes. All scale bars represent 20 mm.

**S5 Fig.** Case 1: Photoacoustic maximum intensity projections (MIPs) in two planes (coronal (top) and transverse (bottom)), at two illumination wavelengths of the contralateral healthy breast. All scale bars represent 20 mm.

**S6 Fig.** Case 3: Photoacoustic maximum intensity projections (MIPs) in two planes (coronal (top) and transverse (bottom)), at two illumination wavelengths of the contralateral healthy breast. All scale bars represent 20 mm.
