# Supplementary figures and images for: Imaging breast malignancies with the Twente Photoacoustic Mammoscope 2
Source: PLoS One. 2023 Mar 2;18(3):e0281434. doi: 10.1371/journal.pone.0281434 (PMC9980787; doi:10.1371/journal.pone.0281434)

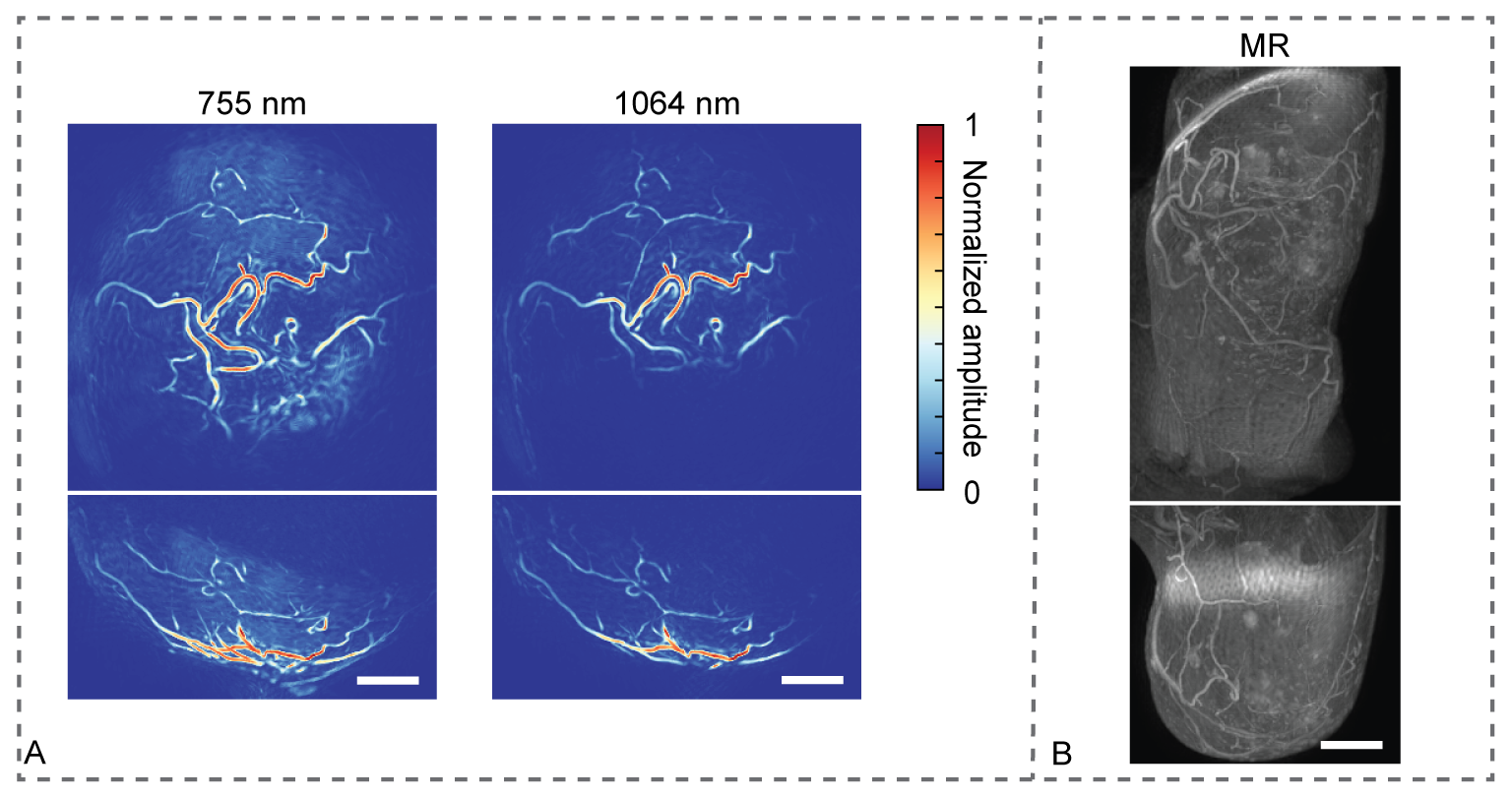

Supplement: S1 Fig — (A) Photoacoustic maximum intensity projections (MIPs) in two planes (coronal (top) and transverse (bottom)), at two illumination wavelengths. (B) Post-contrast dynamic T1 MR MIPs in the same two planes. All scale bars represent 20 mm. (TIF) [file pone.0281434.s002.tif]

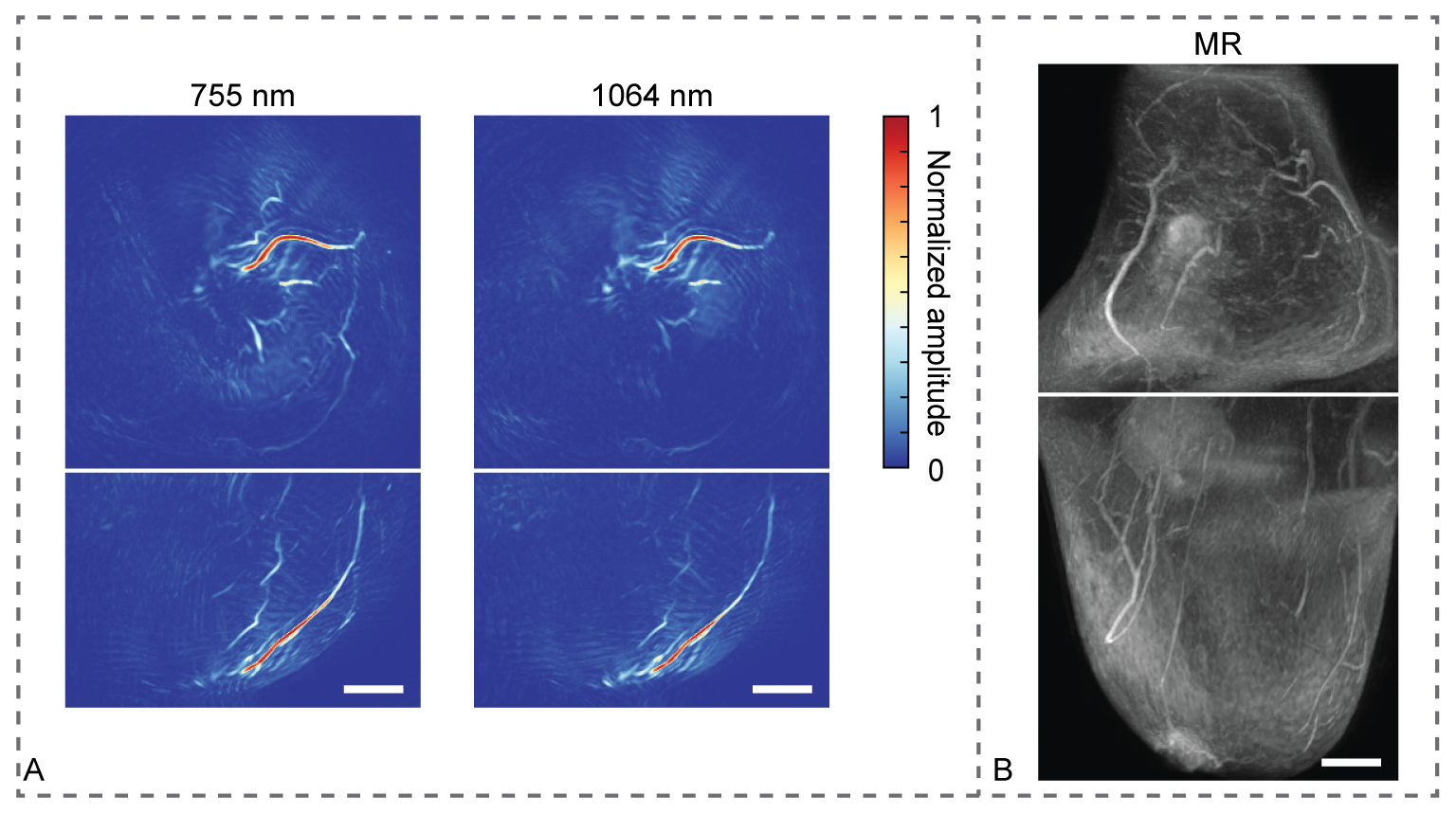

Supplement: S2 Fig — (A) Photoacoustic maximum intensity projections (MIPs) in two planes (coronal (top) and transverse (bottom)), at two illumination wavelengths. (B) Post-contrast dynamic T1 MR MIPs in the same two planes. All scale bars represent 20 mm. (TIF) [file pone.0281434.s003.tif]

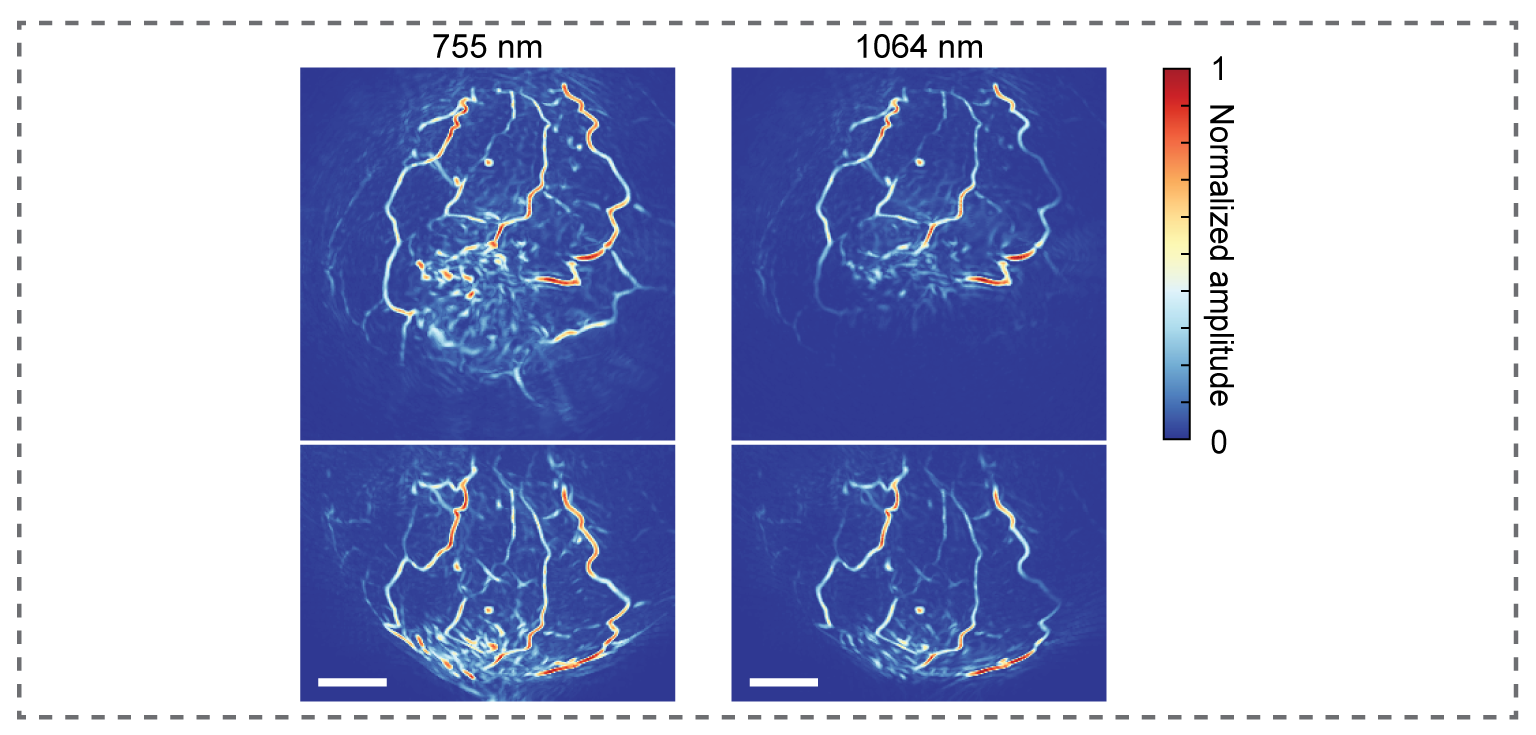

Supplement: S3 Fig — All scale bars represent 20 mm. (TIF) [file pone.0281434.s004.tif]

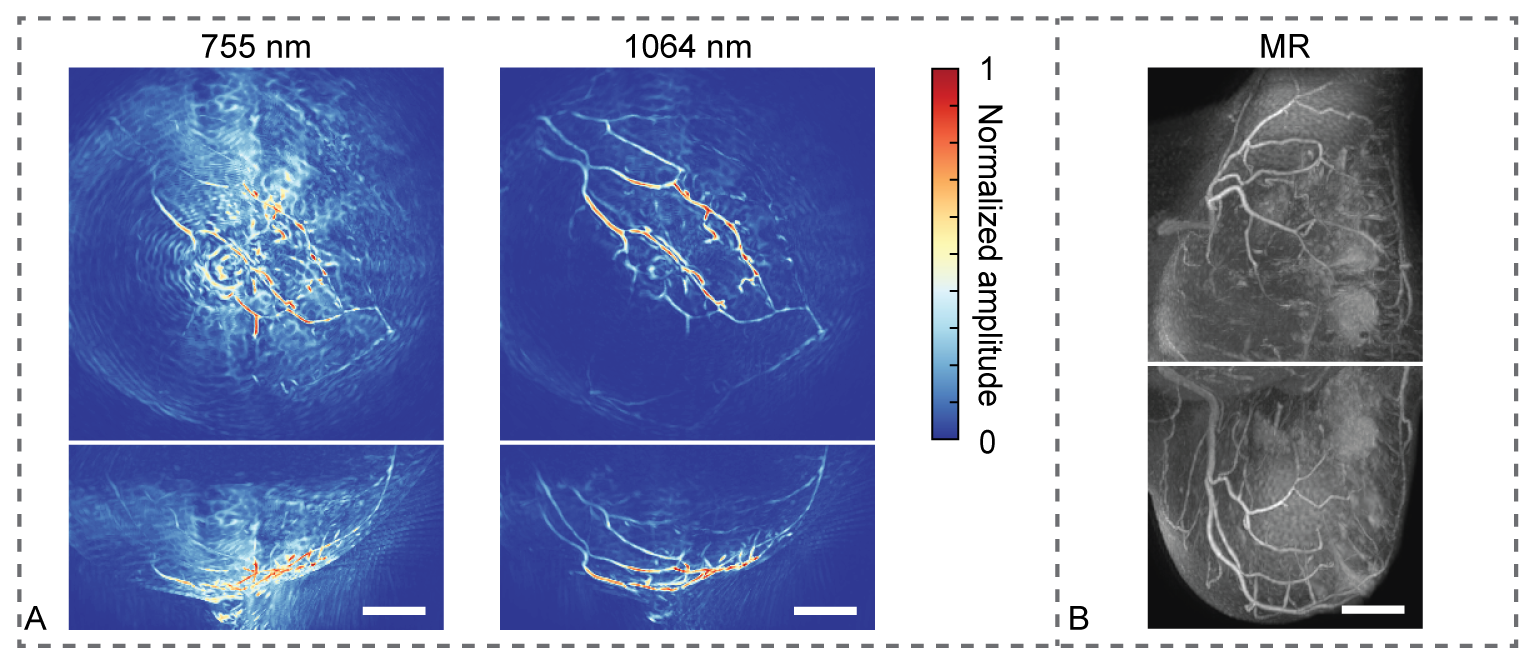

Supplement: S4 Fig — (A) Photoacoustic maximum intensity projections (MIPs) in two planes (coronal (top) and transverse (bottom)), at two illumination wavelengths. (B) Post-contrast dynamic T1 MR MIPs in the same two planes. All scale bars represent 20 mm. (TIF) [file pone.0281434.s005.tif]

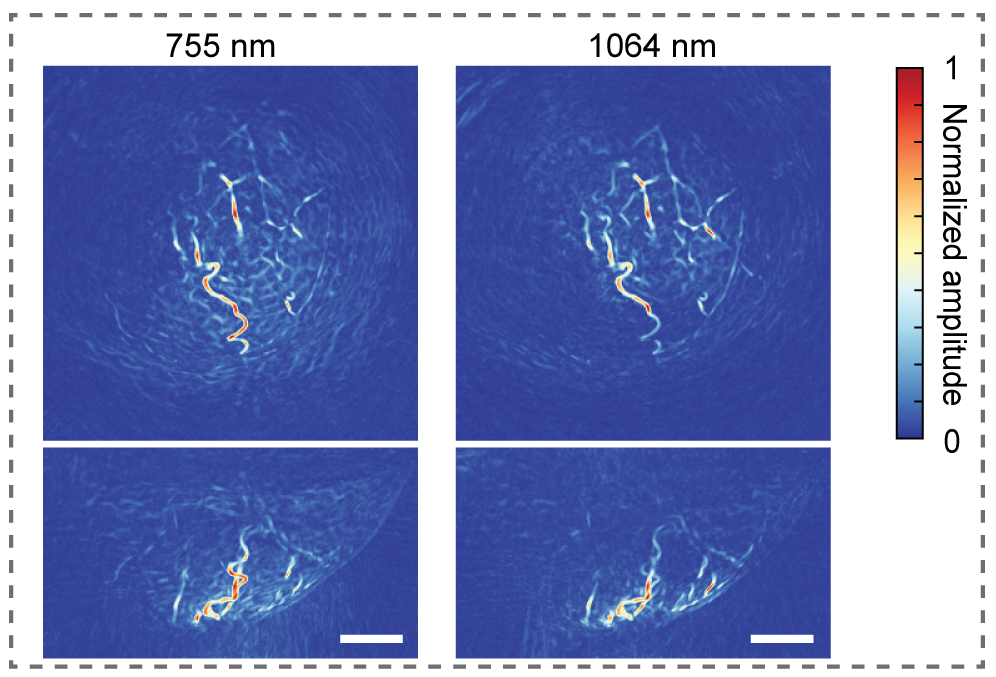

Supplement: S5 Fig — All scale bars represent 20 mm. (TIF) [file pone.0281434.s006.tif]

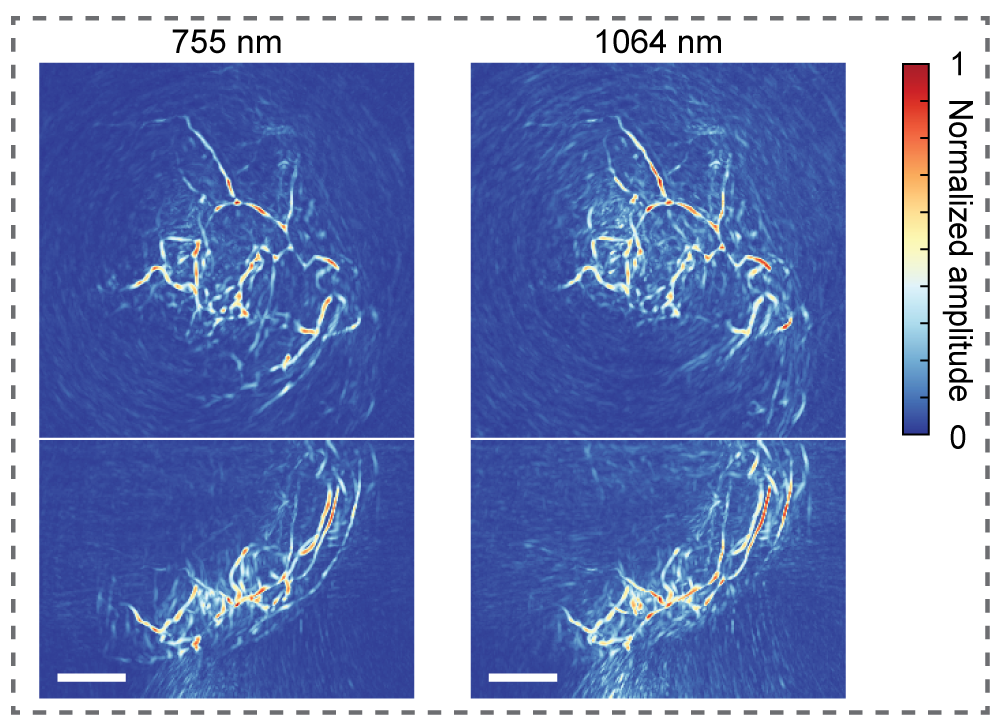

Supplement: S6 Fig — All scale bars represent 20 mm. (TIF) [file pone.0281434.s007.tif]
