# Supplementary material for: Imaging breast malignancies with the Twente Photoacoustic Mammoscope 2
Source: PLoS One. 2023 Mar 2;18(3):e0281434. doi: 10.1371/journal.pone.0281434 (PMC9980787; doi:10.1371/journal.pone.0281434)
Supplement: Attachment — Submitted filename: Response to Reviewers.docx [file pone.0281434.s008.docx]

**Response to reviewer comments on:**

**Imaging breast malignancies with the Twente Photoacoustic Mammoscope 2**

S. M. Schoustra, B. De Santi, T. J. P. M. op ’t Root, C. A. H. Klazen, M. van der Schaaf, J. Veltman, W. Steenbergen, & S. Manohar

We thank both reviewers for the time and effort for a comprehensive review of our manuscript. In response to their questions, comments and observations, we have developed this response, and where necessary and possible, made revisions to the manuscript. We believe the contribution of the reviewers has strengthened this manuscript by removing any unintended ambiguity.

**Reviewer #1:**

**The authors reported clinical measurements of breast cancer patients with the 3D Twente photoacoustic mammoscope prototype. Specifically, 30 patients in total were scanned and 4 out of 19 malignant cases were closely investigated. Photoacoustic imaging results were qualitatively compared with craniocaudal X-ray images, ultrasound images, and MRI images. Even though a larger cohort is expected for more convincing conclusions, the scientific findings of the four cases are of interest to this journal.**

Thank you for your appreciation of our study.

**The following aspects can be improved before further consideration of publication:**

**1. One caveat of the study is that there is no control group. It is unclear how different images will look like in benign cases, especially in PA images. Authors should at least analyze some benign cases and show the X-ray, MRI, US, and PA images for comparison**.

The main goal of the work has been to explore a first set of vascular or vascular-derived features that are associated with breast cancer using a specific imaging geometry. This has been the reason to restrict the inclusions to suspect breasts.

We do understand the requirement to show the appearance of benign cases in comparison. In future, with an improved system, a full breast cancer diagnostic-feature set will need to be developed where it will be crucial to include confirmed benign lesions in addition to malignant lesions.

We have added the following sentence in the ‘Future work’ paragraph of the Discussion:

To develop a full diagnostic-feature set, studies will be performed on a larger dataset which will include benign cases for comparison.

**2. The authors claim that “to enhance signals coming from the deeper vessels, adaptive intensity modulation was applied”. However, from the justification as well as results shown in figures (such as Fig 6), it seems that adaptive intensity modulation does not compensate for low illumination in deep regions. Instead, it adjusts image intensity with respect to local variability, which is more anatomy dependent rather than depth dependent. Please elaborate or correct.**

The reviewer is right that the adaptive intensity modulation (AIM) is indeed used to improve homogeneity of image intensities in regions with low local variability resulting in improved contrast. It was observed that AIM resulted in a selective improvement in the visualization of deeper vessels. The reason is that deeper lying tissues exhibit lower local variability due to lower fluence, lower energy deposition and consequently a lower PA signal. In these situations the AIM amplifies intensity from the target absorbing regions namely the blood vessels. The opposite effect is observed in the superficial vessels which show high local variability values (due to higher fluence and stronger PA signals) resulting in attenuation of their intensity in the output image.

Figure R1 shows the Frangi filtered image and the output of the AIM for case 1 as maximum intensity projections. The most evident effect is the increased homogeneity of the vessel intensity values and the enhancement of structures in the image which were poorly visible in the Frangi filtered image.

*Figure R1. The effect of adaptive intensity modulation on the maximum intensity projections of a PAM 2 patient reconstruction (Case 1). All scale bars represent 20 mm.*

*Figure R2. The effect of adaptive intensity modulation on local (1.5 cm thick tissue slabs) maximum intensity projections (sagittal view). Orange arrows indicate deeper vessels which were enhanced after the application of the adaptive intensity modulation. All scale bars represent 20 mm.*

Figure R2 shows the maximum intensity projections of the Frangi filtered and the AIM output in the sagittal plane of a 1.5 cm thick tissue slab located medially in the breast. The local effect of the AIM on some vessels directed toward the center of the breast can be observed as their contrast and visibility is improved after its application.

Since this aspect needs to be systematically investigated further, we have decided to go by the advice of the reviewer and modify the sentence in the manuscript from:

To enhance signals coming from the deeper vessels, adaptive intensity modulation was applied.

to:

To further improve image contrast, adaptive intensity modulation was applied.

**3. To compensate for depth dependent PA intensity, optical fluence compensation is necessary, which is missing in this work. There have been multiple works on optical fluence compensation for breast. Suggest authors at least implementing a simple model-based method to consider exponentially attenuated light fluence given the boundary of the breast.**

We thank the reviewer for raising this important point. We have indeed studied fluence compensation but decided not to apply to the patient data due to the addition of noise and inaccuracies to the images. Here we demonstrate the negative effects on the images in a favourable case of a breast immobilized with a cup as in Ref. [1] (subject 2), and using a recent and sophisticated method [2] for fluence correction.

The method [2] adopts an automatic and data-driven approach to adaptively estimate the decay factor in the tissue, in contrast to simple models that use assumed knowledge of the average optical properties of the breast. The following steps are implemented:

1. Compensation for non-uniform incident optical fluence: after assuming a spherical coordinate system with the origin corresponding to the breast cup center, the maximum voxel brightness is extracted at each polar angle and fitted with a polynomial curve. Finally, the obtained fitting curve is used to compensate the non-uniform incident optical fluence.
2. Estimation of the breast surface and depth
3. Compensation for the effective optical attenuation: the maximum voxel brightness is extracted at discrete intervals of increasing depth and fitted with an exponential curve based on the Beer-Lambert law. The estimated pre-factor and effective optical attenuation factors are used to compensate for depth.

Figure R3(b) and R3(c) show the estimation of the incident optical fluence distribution and effective optical attenuation, respectively. The final optical fluence compensated images are shown in Figure R3(d).

The curves (1^st^ order polynomial for incident optical fluence, and exponential for effective optical attenuation) fitted the measured data correctly. However, the final result of the compensation is not acceptable. The most prominent problem is noise enhancement in the deepest regions of the breast making the final image difficult to interpret.

It should be noted that unlike the breast in the above exercise, the breasts of patients included were not supported using the cup system. This renders the implementation of fluence compensation even more complicated due to the difficulty in segmenting the breast tissue in the image. Inaccuracy in the breast segmentation impacts the estimation of the effective optical attenuation and will further affect the final images further.

For these reasons, we decided to avoid the use of fluence compensation at this stage of the study, and to apply only Frangi vesselness and contrast enhancement filtering to improve image interpretability.

*Figure R3. Results of optical fluence compensation* [2] *on reconstructed data from a cup-supported breast: (a) Coronal and sagittal MIPs of the original initial pressure reconstructions, (b) maximum voxel brightness at polar angles (solid line) and second-order polynomial fitting (dashed line), (c) maximum voxel brightness at depth (solid line) and Beer-Lambert law based exponential fitting, (d) coronal and sagittal MIPs after optical fluence compensation. All scale bars represent 20 mm.*

**4. Also in image processing, why are the final images obtained by summing the adaptive modulated image and the median filtered image, instead of applying the adaptive modulation directly on the filtered image?**

Figure R4 shows the scheme of the pre-processing pipeline we used, adapted from [3] and [4]:

*Figure R4: Photoacoustic image processing pipeline*

While in Refs. [3] and [4] a 4D transform-domain collaborative filtering [5], we preferred median filtering as a first denoising step for its simplicity.

Summing the adaptive intensity modulated and the median filtered image allows to preserve also potential significant non-vessel like structures in the final image, previously filtered out from the Frangi filtering.

**5. It is difficult to correlate different modalities without registration. The authors discussed the difficulty of cross-modality registration due to a lack of anatomical landmarks, but also mentioned a previous work where MRI and PA images are deformably registered and fused in reference [7] in Introduction. Why not applying the same technique to register at least MR and PA images?**

We agree with the reviewer that image registration is important for any inter-modality comparison. However, we found it highly challenging to register the PA images obtained with our instrument to the MRI images. In Ref [6] (Ref. 7 in manuscript), the authors manually identified common landmarks (e.g., branching points) in both modalities and used them to estimate a thin plate spline deformation model. Using this method, one must find a high number of anatomical landmarks evenly distributed throughout the breast volume in order to obtain an accurate co-registration of the two modalities. We observed large and highly non-rigid deformations that made the task of identifying a sufficient number of common landmarks in PAM2/MR image pairs very difficult; more often only major vascular structures are comparable. Further, some lesions are located in regions with low illumination where the number of identifiable branching points is not sufficient, and finding their correspondence in the MRI images is not straightforward. We believe that at this stage, introducing a registration approach would not improve the comparison between PAM2 and MR images and that the visual examination of slab maximum intensity projections in both modalities is, for now, the choice we are most confident of.

To better clarify this point, we modified the sentence in the Discussion section of the manuscript from:

This was not possible with the data presented in this work, due to a lack of landmarks, e.g. common features.

to:

This was not possible with the data presented in this work, due to a lack of a sufficient number of landmarks identifiable in both modalities, especially in ‘dark’ regions of the image.

**6. In Materials and Methods, the authors mentioned that two US probes were used in the study, i.e. an L12-5 50 and an L18-5. However, only one ultrasound image was shown for each case in Results. Please elaborate the difference of the images acquired from the two probes, and how they were used in diagnosis.**

Both probes are used in US examinations of the breast with the described system. In practice, the radiologist starts the examination with one of the probes and with a certain frequency. Depending on whether the tissue and lesion are clearly imaged, he/she changes probe and/or center frequency.

In general, the L18-5 probe has a slightly broader frequency range (from 18 to 5 MHz) than the L12-5 probe (12 to 5 MHz) and can therefore be used when looking for higher resolutions and/or when superficial structures are to be imaged. In the four cases presented in this work, the presented US images were all obtained with the L12-5 probe.

In order to clarify this point also in the manuscript, we changed the sentence from:

US imaging was done with a Philips EPIQ 5G system (Koninklijke Philips N.V., Amsterdam, The Netherlands), with the L12-5 50 mm linear array transducer and the L18-5 broadband linear array transducer.

to:

US imaging was done with a Philips EPIQ 5G system (Koninklijke Philips N.V., Amsterdam, The Netherlands), with the L12-5 50 mm linear array transducer and/or the L18-5 broadband linear array transducer, depending on tissue and lesion characteristics and radiologist’s experience.

**7. How does the entropy map look like for the other three cases?**

We report here the entropy maps for the 3 remaining of the 4 patients selected in the study. There are some points to be noted before analysing the results. First, as mentioned in the manuscript, for some of these cases it was difficult to confidently trace the the tumor ROI. Second, some reconstructions can suffer from low and inhomogeneous illumination, which is why, it was necessary to perform a custom fine-tuning of the parameter for the correction of the entropy map to properly weigh the influence of the vesselness response on the final entropy map (the parameter is named $k$ in the shared *local_entropy.m* script).

Figures R5, R6 and R7 show the entropy map results of Case 1, Case 2 and Case 4, respectively. In general, the performance of these maps in identifying the tumor is not acceptable at this stage, since the data is affected by inhomogeneities in illumination and the presence of reconstruction artefacts. Also, their validation is still inadequate due to the difficulty in the definition of the ROIs. For example, in Case 1 (Figure R5), it is possible to identify the ROI in the sagittal plane with a fair level of confidence, thanks to the particular geometry of some vessels, but this is not the case in the axial plane. Although in that region it is possible to observe an increase of the entropy at a wavelength of 755 nm, it is not the only region with high level of entropy. In Case 2 (Figure R6), we observe high levels of entropy which correlate with the locations of certain reconstruction artefacts. Nevertheless, the suspected tumor region sees an increase in the level of entropy especially in the map at 1064 nm. This behavior is attributable to the presence in the image of spotty structures possibly attributable to the smaller tumor-related vessels. Finally, in Case 3, the obtained entropy maps produced are characterized by poor quality and difficult interpretation given the high level of noise caused by reconstruction artefacts (Figure R7).

Given the low interpretability of these maps we decided to report only the most promising entropy map so far with the intention to further refine this technique (make it more robust to the noise and illumination level of the region) and applying it in a future study with a larger dataset.

*Figure R5. Photoacoustic maximum intensity projections (MIPs) and entropy maps in two planes, at two wavelengths for Case 1. (A)-(B) In the transverse plane. (C)-(D) In the sagittal plane. All scale bars represent 20 mm. (E)-(F) Post-contrast dynamic T1 MR MIPs in the axial and sagittal planes. The main breast lesion is identified by the blue dashed circle. All scale bars represent 20 mm.*

*Figure R6. Photoacoustic maximum intensity projections (MIPs) and entropy maps in two planes, at two wavelengths for Case 2. (A)-(B) In the transverse plane. (C)-(D) In the sagittal plane. All scale bars represent 20 mm. (E)-(F) Post-contrast dynamic T1 MR MIPs in the axial and sagittal planes. The breast lesion is identified by the blue dashed circle. All scale bars represent 20 mm.*

*Figure R7. Photoacoustic maximum intensity projections (MIPs) and entropy maps in two planes, at two wavelengths for Case 4. (A)-(B) In the transverse plane. (C)-(D) In the sagittal plane. All scale bars represent 20 mm. (E)-(F) Post-contrast dynamic T1 MR MIPs in the axial and sagittal planes. The main breast lesion is identified by the blue dashed circle. All scale bars represent 20 mm.*

**8. In discussion, the authors explained that the oxygen saturation was not evaluated due to the difference in illuminated tissue with the two wavelengths. This is confusing, why would the illuminated tissue change at different wavelengths? Please clarify.**

In the PAM 2 device, a dual-laser system (Qcombo, Quanta System, Milan, Italy) comprising an Nd:YAG and an Alexandrite Q-switched laser is used. In double-wavelength mode, the system alternates between the two wavelengths at a 20 Hz repetition rate [7]. Using two different lasers inevitably leads to differences in the distribution of light between the two wavelengths. This aspect was studied in detail using an experiment and simulation. For the former, a set of the breast-supporting cups were spray painted white and placed in the imaging tank and visible-light photographs were acquired from above the imaging aperture during experiment to visualize the light distribution on the cup for a stationary imaging tank. Figure R8 shows the photographs for two different cup sizes and both laser wavelengths emitted by the PAM 2 system. It can be seen that the beam shape and profiles as well as divergence angles for 1064 and 755 nm are not the same (some intensity differences can also partially be attributed to the lower sensitivity of the camera to 1064 nm than to 755 nm).

| Cup size 6, 755 nm | Cup size 6, 1064 nm | Cup size 8, 755 nm | Cup size 8, 1064 nm |
| --- | --- | --- | --- |
|  |  |  |  |
| *Figure R8. Photographs of light profile on white spray painted cups with different sizes.* | | | |

The result is that the exposure on the breast at the two wavelengths is not identical. This influences the light diffusion in the breast so that certain volumes of the breast for the two wavelengths do not overlap. This makes accurate quantification of oxygen saturation very challenging.

**9. The discussion of improvements achieved from this work compared with the previous generation of Twente mammoscope [5, 6] is inadequate.**

Continuing research on photoacoustic mammography, the second generation Twente Photoacoustic Mammoscope (PAM 2) was developed, where some of the limitations of PAM 1 were overcome. PAM 1 had a limited field of view (90 × 80 mm^2^ detector surface area), made use of breast compression and the flat 2D detector array complicated 3D visualization of lesions.

We removed the following sentence in the manuscript:

PAM 2 is a tomographic system, illuminating and detecting ultrasound from around the breast.

And added the following sentences in the manuscript to further discuss the improvements of the PAM 2 device compared to PAM 1:

In PAM 2 the breast is pendant in water without any compression, with illumination and ultrasound detection from around the breast. Illumination is performed with 10 beams (as opposed to one large beam in PAM 1), where one beam is directed at the ventral side of the breast, and nine smaller beams directed at the region of the breast close to the chest wall. Further, illumination can be provided at 2 wavelengths 755 and 1064 nm (as opposed to single-wavelength illumination at 1064 nm in PAM 1). The ultrasound detectors are arranged as 12 arcs with 32 elements each around the breast; the arc-shapes follow the pendant breast contour.

**10. The discussion can be organized more systematically and concisely by concluding all cases by their features, instead of listing one by one again as in Results. Suggest authors using “can be” instead of “might be” or “seems that” to conject.**

We thank the reviewer for these suggestions. We reorganized the Discussion section following the reviewer’s advices.

**Reviewer #2:**

**The manuscript verified the clinical feasibility of PAM-02 system to diagnose breast cancer. Overall the manuscript is acceptable to publication. Please address the minor question of the PAM-02 system.**

Thank you for your appreciation of our study.

**The authors used 795nm and 1064nm lasers for imaging. Please justify why both wavelength are chosen and other research (10.1038/s41467-018-04576-z) stated single 1064 laser was used to detect breast cancer, why did you use dual-band laser?**

Indeed the work described in Ref. [8], has used a single wavelength at 1064 nm to extract vascular information which was used to identify breast cancer. The motivation to additional wavelengths is to perform quantitative photoacoustic imaging [9] which has the goal to extract estimates of chromophore concentrations in tissue, such as hemoglobin or lipids, since these chromophores have wavelength dependent optical absorption [9]. The use of two appropriately chosen wavelengths can help to calculate a measure of blood oxygen saturation (SaO_2_), which is a subset problem in quantitative photoacoustics, given that SaO_2_ is a ratio. Knowledge of SaO_2_ will provide an assessment of tissue metabolism, known to be dysregulated in cancer, and will potentially provide a means to discriminate between benign, pre-invasive and malignant lesions.

The ability to recover the blood oxygenation saturation at a suspicious site in the breast is one of the challenges in the field. For this accurate fluence compensation approaches, are crucial to making accurate estimations which has been challenging to implement (see also reaction to Comment 3 of Reviewer 1 on pg. 3-4-5).

**References:**

[1]	S. M. Schoustra, T. J. P. M. op ’t Root, R. P. Pompe van Meerdervoort, L. Alink, W. Steenbergen, and S. Manohar, “Pendant breast immobilization and positioning in photoacoustic tomographic imaging,” *Photoacoustics*, vol. 21, p. 100238, Mar. 2021, doi: 10.1016/j.pacs.2020.100238.

[2]	S. Park, F. J. Brooks, U. Villa, R. Su, M. A. Anastasio, and A. A. Oraevsky, “Normalization of optical fluence distribution for three-dimensional functional optoacoustic tomography of the breast,” *J Biomed Opt*, vol. 27, no. 03, Mar. 2022, doi: 10.1117/1.jbo.27.3.036001.

[3]	L. Lin *et al.*, “High-speed three-dimensional photoacoustic computed tomography for preclinical research and clinical translation,” *Nat Commun*, vol. 12, no. 1, pp. 1–10, Feb. 2021, doi: 10.1038/s41467-021-21232-1.

[4]	L. Lin, X. Tong, P. Hu, M. Invernizzi, L. Lai, and L. v. Wang, “Photoacoustic Computed Tomography of Breast Cancer in Response to Neoadjuvant Chemotherapy,” *Advanced Science*, vol. 8, no. 7, pp. 1–10, 2021, doi: 10.1002/advs.202003396.

[5]	M. Maggioni, V. Katkovnik, K. Egiazarian, and A. Foi, “Nonlocal Transform-Domain Filter for Volumetric Data Denoising and Reconstruction,” *IEEE Transactions on Image Processing*, vol. 22, no. 1, pp. 119–133, Jan. 2013, doi: 10.1109/TIP.2012.2210725.

[6]	M. Toi *et al.*, “Visualization of tumor-related blood vessels in human breast by photoacoustic imaging system with a hemispherical detector array,” *Sci Rep*, vol. 7, no. 1, p. 41970, Feb. 2017, doi: 10.1038/srep41970.

[7]	S. M. Schoustra *et al.*, “Twente Photoacoustic Mammoscope 2: system overview and three-dimensional vascular network images in healthy breasts,” *J Biomed Opt*, vol. 24, no. 12, p. 1, 2019, doi: 10.1117/1.jbo.24.12.121909.

[8]	L. Lin *et al.*, “Single-breath-hold photoacoustic computed tomography of the breast,” *Nat Commun*, vol. 9, no. 1, p. 2352, Dec. 2018, doi: 10.1038/s41467-018-04576-z.

[9]	Y. Zhou, J. Yao, and L. v. Wang, “Tutorial on photoacoustic tomography,” *J Biomed Opt*, vol. 21, no. 6, p. 061007, 2016, doi: 10.1117/1.jbo.21.6.061007.
