# Supplementary material for: Imaging breast malignancies with the Twente Photoacoustic Mammoscope 2
Source: PLoS One. 2023 Mar 2;18(3):e0281434. doi: 10.1371/journal.pone.0281434 (PMC9980787; doi:10.1371/journal.pone.0281434)
Supplement: Attachment — Submitted filename: PONE-D-22-20783_YWu_R2.docx [file pone.0281434.s009.docx]

**Partially addressed:**

Comment 1: Let alone the benign cases for now, is it possible to show images of normal breasts so that readers can have a clearer sense how the vascular features are different in breast tumors from normal breasts?

**Suggestions**:

Comments 2, 3, 4, 5, 7 are well addressed. If the authors choose to publish the peer review history then it might be fine. Otherwise, the revised manuscript is a bit too simplified. If the authors choose not to publish the peer review history, suggest authors include the analysis and justifications that you responded to the rebuttal to the Discussion Section to make the manuscript more comprehensive. Figures R2, R3, R5, R7 should be added to Supporting Information. Figure R4 should be added to Methods.
